# Conference on Railway Sanitation

**Published:** 1904-03

**Authors:** 


Conférence on Railway Sanitation.—A number of chief surgeons and superintendents of Texas railroads met in the office of State Health Officer Tabor in Austin, February 23d, to talk over Dr. Tabor's recent rules and regulations to govern railroad disinfection and general sanitation, in pursuance to the recently passed law on the subject. These rules and regulations were published in our January number. Amongst the surgeons were Dr. W. G. Jameson, Chief Surgeon of the I. & G. N. ("The Texas Railroad"), the only road, bye the bye, on which disinfection of cars is in operation; Dr. R. W. Knox, Chief Surgeon S. P. System; Dr. J. R. Stewart, of the H. & T. C.; Dr. C. A. Smith, of the Cotton Belt, and Dr. Scott, of the G., C. & S. F. Mr. Pettybone, Superintendent of the G., C. & S. F.; Mr. Van Vleck, Superintendent of the S. P. System, were here also. The date when the rules become effective is March 31st, as heretofore announced.

I can not see that anything was accomplished by the conference. I was not present, but, from the press reports, it seems that there was a general protest against having spittoons in the ladies' cars, as tending to encourage chewing in these cars. It was urged that cars entering Texas from other States, where there is no law to require it, are not provided with spittoons, and, as Texas lines take these cars at the State lines in some instances, they become liable for failure to provide them. Dr. Knox also thought "disinfection" of cars, after they enter Texas, would be unnecessary, if they were properly disinfected at point of departure, St. Louis, for instance. There is no disinfection of cars done at St. Louis, New Orleans or elsewhere, so far as I know. It is required by law only in Teaxs. Hence, that objection is not valid. Besides, the law regarding disinfection of coaches, and Dr. Tabor's rules to enforce it, are very vague and indefinite. They are to be disinfected "when they become infected." Now, who is to be the judge, and say when a day coach is infected? While with regard to sleepers, they are to be disinfected only "at the end of each run." It may be assumed that, as some of the roads protest against the enforcement of these rules, and object to doing more than the law requires, the disinfection, if practiced at all, will be of a perfunctory nature, and only sufficient to comply with the requirements and save themselves. It is to be assumed that cars in which consumptives travel are always infected, and Dr. Tabor should, and doubtless will, see that the disinfection is thorough and effective, or it will not be done. The railroads—some of them—are very much of Jay Gould's opinion, "the public be damned." They are out for revenue. Dr. Tabor will not prescribe the manner of disinfecting, nor the apparatus to be employed. It is generally understood, however, that formaldehyde must be used, as it is the only disinfectant suitable for sleepers, and at once effective and free from objection. Whatever plan or machine adopted by any road, must, however, have his approval.

To effectually fumigate a car in which tubercle bacilli are supposed to be lodged in the fabrics, formaldehyde gas should be used in vast volume, so as to penetrate. The use of sheets wet with formaline solution will not disinfect a car. It is, at best, only a deodorizer.

